# Constructing the festival tourist attraction from the perspective of Peircean semiotics: The case of Guangzhou, China

**DOI:** 10.1371/journal.pone.0282102

**Published:** 2023-02-21

**Authors:** Jing Li, Gouxiong Yu

**Affiliations:** 1 School of Foreign Languages & International Business, Guangdong Mechanical and Electrical Polytechnic, Guangzhou, China; 2 School of Business Administration, Guangdong University of Finance, Guangzhou, China; University of Naples Federico II: Universita degli Studi di Napoli Federico II, ITALY

## Abstract

Based on the Peircean semiotic theory, this study aims to explore the semiotic construction of a festival tourist attraction using the Guangfu Temple Fair in China as a case. A qualitative research method of grounded theory was used to analyze the organizers’ planning scheme, conference materials, 7 interviews, and 45 tourists’ interviews. We found that festival organizers produce festivalscape based on social values and tourists’ expectations, including safety assurance, cultural activity, personnel service, facilities, creative interaction, food, a trade show, and the festival atmosphere. Tourists in the festivalscape, through cultural, novel, social, and emotional experience and collateral observations, assign meaning to the festival’s attractiveness in terms of cultural diversity, vibrant activities, distinctive features, and a sense of ceremony. These findings indicate that organizers’ sign production and tourists’ sign interpretation constitute the conceptual model of the semiotic construction of festivals as tourist attractions. Furthermore, the study extends the understanding of tourist attractions and will help organizers to create successful festival attractions.

## 1. Introduction

Recently, a huge increase in tourists participation has rendered festivals one of the fastest-growing tourist attractions [1]. In fact, festivals become tourist attractions based on tourists’ participatory experiences [2]. Regarding studies on the festival tourism experience, scholars believe that tourists have changed from passive recipients to active constructors [3, 4]. Consequently, the tourism experience is no longer determined by objects provided by practitioners; instead, tourism has evolved into an embodied practice involving subjects and objects, wherein many parties assign meaning to tourist attractions through their participation. This process of meaning construction is also called semiotic construction [5].

The founders of modern semiotics were Saussure and Peirce, who established two distinct theoretical traditions [6]. In contrast, the Peircean semiotic has frequently been tested in terms of its applicability and validity to tourism research (e.g., [7–9]). MacCannell [10] (pp. 109–120) first applied the Peircean semiotic theory to tourism research and proposed a semiotic system of tourist attractions from the perspective of sociology. According to MacCannell, a tourist attraction consists of three parts: a sight, marker (information about scenery), and tourist. Furthermore, he emphasized the prominent position of the marker. Recently, scholars have become aware of sign meanings conveyed in markers—such as postcards [11, 12], tourism brochures [13], photos [14, 15], and site entry tickets [16]—to understand tourism practitioners’ expected experiences as expressed through postcards and tourism advertisements, or tourists’ experiences expressed through photographs. However, one party does not determine the tourism experience; it is a multi-party process of assigning meanings through practices. Therefore, the tourism experience must be taken in its entirety to explore the construction of the meaning of tourist attractions. Acknowledging this, Soica analyzed the semiotic construction of a touristed landscape by studying tourism companies’ advertisements and visitor books [5]. However, as tourist attractions, festivals differ from landscapes; thus, their semiotic constructions will also be distinct.

Festivals provide a platform to showcase the destination’s intangible cultural heritage, local traditions, ethnic backgrounds, and cultural landscapes, and are gradually becoming important tourist attractions for destination promotion [2]. As a product of the human spirit, festivals are characterized by rituals, special atmosphere and services, short duration, high level of personal contact and interaction, crowds, and repeated celebrations over time, which are unmatched by other types of tourism attractions [17]. Festivals are not static; their content, organization, and form change in response to external and internal factors [18]. The success of festivals depends more on the enthusiasm of local communities and organizers than on natural resources or architectural attractions [19] (pp. 789). Tourist attractions possess objective and symbolic properties [20]. A tourism landscape attraction is centered on the tourism landscape object, meaning the attraction’s objective property is relatively fixed. However, a festival tourist attraction is centered on the festivalscape, which is a physical environment including both tangible factors and an ambience for activities [21]. Thus, the objective property of a festival tourist attraction is more malleable, and accordingly, its symbolic property changes once its objective property changes. Organizers can provide a specific experience to tourists by manipulating the festivalscape, while tourists can assign meaning based on their experiences; thus, both participate in assigning meaning to tourist attractions through interactions. Despite thriving festival tourism, festivals, as semiotic constructions of tourist attractions, have not gained sufficient attention in the literature.

Peircean semiotic theory plays a dominant role in tourism research [7], connecting the observable representations of signs to their signified object and emphasizing the interpretant role of signs. Just as Metro-Roland said, the Peircean semiotic theory goes beyond the analysis of concepts and their textual and auditory expressions, and it is considered more appropriate for understanding non-textual objects in social settings as signs [22]. Using Peircean semiotic triangle as a theoretical foundation, this study examines the semiotic construction process of festivals from their inception to their gradual emergence as tourist attractions, using newly created festivals as an example. The research context is based on Chinese festivals, because China has witnessed a remarkably rapid development of diverse festivals, attracting widespread attention from academia [23, 24]. Based on this, we propose the following research questions: 1) How do festival organizers produce signs by creating attractive festivalscapes? 2) How do tourists interpret the meaning of signs in festivalscapes designed by organizers? Since the above research questions are still under exploration, this study adopts the qualitative method of grounded theory to understand the construction process of festival tourist attractions. Grounded theory has been successfully applied to the study of semiotics (e.g., [25]) on the assumption that theories can be systematically established from the analysis of social environments [26]. Although grounded theory has the essential characteristics of qualitative research, its potential in generating new theoretical indirection goes beyond most qualitative methods [27], as it provides unique guidelines for developing theories [28]. The inductive nature of grounded theory makes it a suitable methodological tool for this study, which attempts to expand and develop a conceptual framework based on Peircean semiotics to explain how festival organizers and tourists jointly give meaning to festival attractiveness and thus construct festival tourist attractions.

To achieve the research objectives, this study first examines existing literature on the semiotic construction of tourist attractions and festivals as tourist attractions. Second, based on the Peircean semiotic theory and the features of festivals, an analytical framework for the semiotic construction of festival tourist attractions is provided. Third, through grounded theory, the general schemes of festivals, preparatory meeting records, and interview materials are analyzed. In addition, the study discusses how organizers create festivalscapes to provide tourists with their expected experience. and explores how tourists form an on-site experience when they interact with the festivalscape, thereby explaining the festival’s attractiveness. Next, we build a conceptual model of the semiotic construction of a festival attraction and clarify the process through which multiple parties assign their meanings to festival attractiveness. Finally, we conclude and highlight implications.

## 2. Literature review

### 2.1. The semiotics of tourism

Anything that conveys some meaning can be called a sign. Signs dominate human verbal and nonverbal communication [7], and exist in all corners of our lives. Semiotics is the study of the structure of meaning [6], and the ideas of semiotics are applied to a wide range of social phenomena in multidisciplinary fields such as linguistics, anthropology, sociology, and tourism. Tourism is a typical socio-cultural phenomenon, and from a semiotic perspective, tourism is the search for unique and unusual signs and landscapes [29]. In tourism studies, semiotics is used to describe or explain how specific items are symbolized as tourism attractions, such as daily necessities, landmark buildings, holiday destinations, and cultural phenomena (handicrafts and festivals) [9].

Although Saussure and Peirce simultaneously served as founders of modern semiotics, their semiotic ideas were two different theoretical systems. Saussure focused on linguistic signs, providing a classical semiotic dichotomy. Peirce, on the other hand, extended his study to all things in the world and proposed the semiotic triangle theory [6]. Accordingly, scholars of tourism studies have explained and developed tourism phenomena based on these two different systems of semiotic theory. One is represented by scholars such as Culler, who adopted Saussure’s semiotic approach to the study of tourism [29], focusing mainly on the symbolic nature of signs. The second is represented by MacCannell et al. [10], who used Peircean semiotics to study tourist attractions, focusing mainly on the social construction process of signs. This study uses Peircean semiotic triangle framework to interpret festival tourism attractions as signs as a combination of "representamen/object/interpretant".

A tourist attraction is a kind of objective existence, but also the product of semiotic construction [20]. MacCannell first introduced semiotics into the field of tourism. Influenced by the Peircean semiotic theory, he considered tourist attraction a sign, and the attractiveness of a tourist attraction is a ternary relationship formed between sight, markers, and tourists. Furthermore, MacCannell proposed semiotic attraction theory, which deals with the semiotic construction of tourist attraction as sight sacralization in five stages: naming, framing and elevation, enshrinement, mechanical reproduction, and social reproduction [10] (pp. 44–45). His research provided two frameworks for the semiotic construction of tourist attractions, used in further research. For example, using semiotic attraction theory, Jacobsen elaborated on the process of the North Cape’s gradual sacralization as a tourist attraction but provided no theoretical breakthrough [30].

Inspired by the research on the ternary relationship of tourist attraction, Metro-Roland believed that the Peircean semiotic theory provides a convincing theoretical explanation of how tourists understand their surrounding environment [31]. Based on this and using the cultural tourism experience in Budapest as an example, Metro-Roland explained how urban tourism is intricately linked with urban activities and places and how tourists experience cultural tourism by reading about the signs of urban sights [22] (pp.11-17). Soica highlighted the relationship between tourism and landscape, defining tourism as a meaning construction. Using the Peircean semiotic theory and Barthes’ signifying system, he examined the construction of a touristed landscape through the websites of two ecotourism companies and books written by tourists. Soica then introduced the semiotic framework regarding tourism as a practice of meaning construction [5].

Both Saussure’s and Peircean semiotics make it possible to interpret signs with great analytical precision, but Peircean theory is more applicable to explaining the semiotic construction of festival tourism attractions for the following reasons: 1) Unlike Saussure’s linguistic-based semiotics, Peircean concept of signs stems from his pragmatic philosophy, and it is more applicable to explaining how tourists perceive objects in their physical environment, society and culture. 2) Peircean basic framework introduces the interpretant directly into the semiotic definition, rather than just emphasizing the sign itself, and it is more in line with the importance of active tourist participation in the experience in modern tourism. 3) Peircean analytical framework helps analyze the socio-cultural operating mechanism behind the sign and can reveal more clearly the process of social construction of the sign meaning of the festival as a tourist attraction.

### 2.2. Festivals as tourist attractions

Falassi described a festival as a social occasion occurring periodically, wherein participants share their worldviews in a context of different nationalities, languages, religions, and histories [32] (pp. 2). Festivals not only protect local cultural traditions and enhance social cohesion, but also promote the economic, social and cultural development of the destination [33], which has become one of the fastest growing parts of tourism [34]. Tourist destinations around the world are competing to capitalize on local artistic and cultural resources to host festivals; in China, for example, the number and type of festivals have grown tremendously since the 1990s, with more than 5,000 places hosting festivals each year today [35]. From a tourism perspective, festivals are important because they are rooted in the attractiveness of a particular region to attract tourists [36]. Festivals have become the crucial source of income for cultural facilities, organizers, and destinations [37] (pp. 35–62), and as Litvin et al. confirm, the increased tourism revenue during festivals due to higher accommodation prices can significantly boost the economy of local communities [38].

Festival tourism research is a specialized field. One of the basic objectives of this research field is to identify festival characteristics and how to create and manage special experiences for tourists [39]. During festivals, a series of activities are arranged around specific themes to provide tourists with the latest information on the local culture, society, and history [40], thereby creating an experience that may appeal to them. Leiper pointed out that tourists are an essential part of the tourist attraction system, without which tourist destinations cannot be considered tourist attractions [41]. Similarly, festivals will not be tourist attractions without tourists’ participation.

Although countless local festivals are explicitly marketed as tourist attractions and attract large numbers of visitors [24, 39], some scholars argue that tourism-oriented activities labeled as "festivals" actually lack genuine festival quality [42, 43] (pp. 1–31), and linking festivals to tourism may lead to a decline in cultural authenticity [44]. In other words, festivals and tourist attractions do not have a natural link [2], and their connection is based on the interaction between festival activities and tourists. Picard described how Bali Island shaped cultural performance into a tourist attraction. The development of tourism in Bali led to a demand for cultural performance, and in response, residents constructed a cultural representation based on the local culture and their understanding of tourists’ expectations. Therefore, cultural performance is considered a dialogue between tourists and residents, and between the universality of international tourism and particularity of the tourist destination [45]. Ma et al. suggested the importance of festival uniqueness and quality for successful festivals and harshly pointed out that one of the reasons why masses of contemporary festivals in China are not attractive to tourists and local residents is that organizers de-contextualize or mis-contextualize festivals, emphasizing that the design of festival experiences should be based on local historical and geographical contexts [39]. Maeng et al. also emphasized the uniqueness of festivals and believed that the attractiveness of festivals came from the unique festival environment atmosphere and activities, including the special events, atmosphere or environment to enjoy festivals [46].

The combination of modern semiotic ideas and tourism theory has both revealed the nature of tourism phenomena and promoted the development of semiotics. Influenced by the semiotic approach summarized by Echtner [6], tourism scholars are keen on interpreting the meaning of tourism promotional materials (posters, videos, advertisements, etc.) [47, 48]. In recent years, research perspectives and studies have been gradually enriched. Gradually, scholars have used Peircean semiotics to explain tourism phenomena such as landscape tourism attraction construction [5], authenticity [7], place branding [8], and multiple meanings of tourism celebrities [9]. However, almost no scholars have studied festivals from a semiotic perspective, which is contrary to the booming development of festival tourism. Therefore, this study analyzes how festivals become tourism attractions through organizers’ planning and tourists’ participation based on the theoretical framework of Peircean semiotics, to provide some reference for successful planning of festival attraction and attracting a mass of tourists’ participation.

## 3. Theoretical framework

### 3.1. The Peircean semiotic triangle theory

The concept of the Peircean semiotic theory is based on a ternary relationship: object-representamen-interpretant. Object is anything represented by the representamen, and interpretant is another sign developed by the interpreter. The semiosis is the “cooperation” between these three components [49] (pp. 594). The relation between the three components is as follows: (1) Object decides representamen, and the representamen partially or partly reflects the effect of the object and presents the quality or characteristic thereof in an abstract way [50] (pp. 208). (2) The representamen determines the interpretant and can create another equivalent or more developed sign in the interpreter’s mind [50] (pp. 228) without being affected. The interpretant is the interpreter’s cognition of the meaning of the sign and the effect of interpretation on the interpreter, which can be cognitive, emotional, or behavioral response [51]. Therefore, the interpretant is the interpretation of the representamen [49] (pp. 594). This interpretation process requires the interpreter’s collateral observation, which refers to the pre-knowledge of what the representamen signifies, such as the interpreter’s knowledge and social experience before semiosis [52] (pp. 493–496). (3) For these, the representamen establishes a connection between the interpretant and object, and passes the sign meaning of what the object signifies to the interpretant, becoming a physical carrier that connects the two.

### 3.2. Semiotic analysis framework of festival tourist attraction

A festival can be considered a sign in the tourist attraction system [53]. It is a temporary cultural celebration activity and promoter of the experience economy [54] (pp. 31), involving a degree of organizational capacity, and is celebrated periodically over time [55]. The essential attribute of festival attraction is festival attractiveness. Festival organizers strive to explore the potential of local culture and enhance the attractiveness of festivals by designing and planning programs, creating atmosphere and providing facilities. This organic combination of tangible factors and activity atmosphere is called festivalscape [23]. Through on-site experiences in a festivalscape, tourists assign meanings to festival tourist attractions. In short, festival organizers and tourists participate in the semiotic construction of festival tourist attractions.

**Fig 1:**
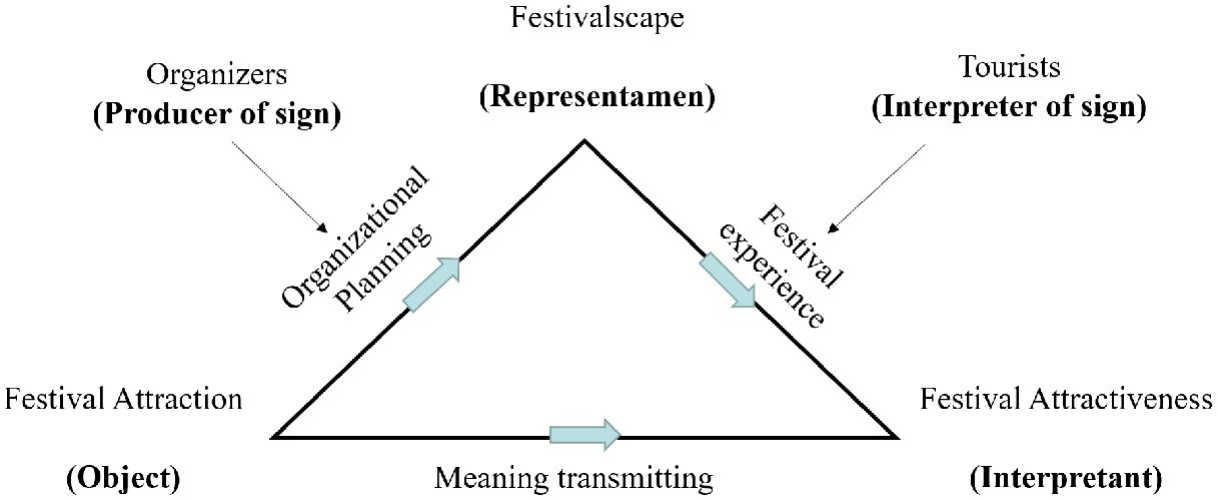
The semiotic analysis framework of festival tourist attraction.

This research applied the interpretation and derivation system in the Peircean semiotic theory to studying the semiotic construction of festival tourist attractions. The analytical framework is as follows. First, the festival attraction is the object of its sign activity, which plays a dominant role of determining the semiosis in general. Organizers use festivalscapes, namely the representamen, to represent the festival attraction. The relationship between the festival attraction and the festivalscapes constitutes a "real causality". Festival organizers play an important role in this relationship by creating attractive festivalscapes that represent the characteristics of festival attraction, thus creating a series of meaningful experiences that attract tourists. Second, nothing is a sign unless interpreted as such [50] (pp. 308); thus, a festival is not a festival tourist attraction unless tourists participate in it, as mentioned. Therefore, tourists’ interpretation of the festival attractiveness is in the position of the interpretant. Through on-site experience in the festivalscapes, tourists can take advantage of collateral observation to understand the sign meaning carried by the representamen. In Peircean opinion, the interpretant is the effect of the representamen on the interpreter, and this can be considered a psychological effect produced by the representamen on the interpreter or as the interpreter’s conditional reflex to the representamen [49] (pp.594). Third, the festivalscape is the carrier that connects the festival attraction and the festival attractiveness. The festivalscape is determined by the organizer’s understanding of the essence of the festival attraction, which in turn determines the interpretation of the festival attractiveness in the tourist’s mind. Therefore, the ternary relationship between festival attraction, festivalscape, and festival attractiveness helps explain the semiotic construction process of the festival as a tourist attraction (as shown in Fig 1).

## 4. Methodology

### 4.1. Grounded theory approach

Grounded theory is one of the most commonly used qualitative methods in social sciences [56] (pp. 359–380), including tourism [57]. This study attempts to establish a conceptual framework to explain the semiotic construction process of festival tourism attractions, and the grounded theory method is considered to be the most appropriate research method. First, the semiotic construction of festival tourism attractions is a relatively new and under-explored topic. Qualitative research methods are suitable for studying such research topics where the internal mechanisms are not yet clear. Second, the process of semiotic construction involving festival organizers and tourists is a "how" and "why" issue, which is inductive and exploratory. The grounded theory approach can provide a valuable tool for in-depth study of the phenomenon in the process [56] and is suitable for explaining the research questions of this process. Third, the process of semiotic construction of festival tourism attractions involves multiple subjects. The method of data collection for grounded theory originates from the research question [58]. The method of continuously collecting new data helps to discover patterns from complex phenomena, facilitate the presentation of interrelationships among multiple constructs, provide new insights into specific phenomena, and generate theoretical propositions [57]. The constructivist grounded theory adopted in this study holds that researchers find new ideas through theoretical review and conversations with data to construct grounded theories. Therefore, this study draws on the grounded theory planning of constructivism to define the semiotic construction process of festival organizers’ sign production and tourists’ sign interpretation.

### 4.2. Case selection

Since the 1990s, the number and types of Chinese festivals have grown tremendously. To some extent, this is a reflection and response to the significant economic and social changes that China experienced during this period. Many cities in China hold festivals to attract tourists, improve their urban image and promote regional economic development [39]. Guangzhou is a famous historical and cultural city in China. To better popularize the Guangfu culture and promote Yuexiu District as “the birthplace of Guangfu culture,” the Yuexiu District Government of Guangzhou city held the first Guangfu Temple Fair in 2011. Since then, the scale of the fair has grown, and the contents have been constantly adjusted, enriched, and innovated. The 9^th^ Guangfu Temple Fair was held between February 19–25, 2019. To attract tourists to the cultural festival, organizers designed a series of thematic activities such as Guangfu cultural and art performances, a talent show, culture symposium, and intangible cultural heritage exhibition. These activities covered culture related to blessings, folklore, food, business, and recreation. According to the statistics of the organizing committee of Guangfu Temple Fair, 45 thematic cultural activities and 279 cultural and art programs and exhibitions were held, and 33 intangible cultural heritage projects were exhibited. The temple fair attracted more than five million visits and generated a turnover of eight million Yuan. Over its nine years of development, the temple fair’s popularity and influence have grown, and it is now a grand annual festive occasion for the Guangfu folk culture.

Guangfu Temple Fair was selected for this study for the following reasons. First, despite being relatively new, the fair has great influence. Thus, analyzing it as a festival provides a more comprehensive discourse on organizers’ efforts in the construction of a festival attraction. Second, the fair integrates traditionality and modernity, and its analysis helps explore tourists’ acceptance of new occurrences. For example, all aspects of the first Guangfu Temple Fair were questioned, from the organization of activities to the arrangement of the contents [59]. After receiving public feedback, the organizers reflected on and improved the event’s organization, striving to gain more acceptance and appreciation. This interactive process helps showcase the participation of many parties in the semiotic construction of festival tourist attractions.

### 4.3. Data collection

Currently, Guangfu Temple Fair is led by the Yuexiu District Government, hosted by Guangfu Temple Fair Organization Committee, and implemented by the Yuexiu District Cultural Development and Promotion Association. At the end of December 2018, the first author contacted the organization committee and was permitted to conduct an internal survey as an intern by participating in the entire event process, which lasted for nearly three months, as follows: (1) During the preparation stage of Guangfu Temple Fair (January 7 to February 18, 2019), we conducted a field survey in the form of participatory observation and semi-structured interviews, and finished preparing the work contents, which included on-site meeting records and preparation of a preliminary publicity draft. With the permission of those in charge, we also collected past years’ materials on Guangfu Temple Fairs, including interviews with the organizers, to more accurately understand the construction of the festivalscape based on attractiveness. The interview design was mainly based on the interview questions in Liang et al. [60] and Ferdinand et al. [61], such as *"What are your reasons for organizing festival*?*" "How do you shape the festival environment to meet the changing needs of tourists*?*" "What factors do you think about when organizing the festival*?" (2) During the implementation of the Guangfu Temple Fair (February 19–25, 2019), we collected materials of tourist interviews from different activity areas. Focusing on the semiotic construction of festival tourist attractions, tourists were asked about their on-site experiences at the temple fair and their understanding of festival attractiveness. The interview questions were designed with reference to Chen’s themes about the festivalscape [23], such as *"How do you feel about the festival*?*" "What do you think of the festival environment*?*" "How is/was your festival experience*?*"* (3) After the 9^th^ Guangfu Temple Fair, we participated in the closing meeting and continued collecting interview data from tourists through the Wechat platform, telephone, and other ways. Interviewees were selected using a theoretical sampling technique to include the perspectives of individuals from diverse socio-demographic backgrounds. Table 1 details the three stages of data collection, and provides interviewees’ profiles. Both before and after the event, this thorough tracking survey could guarantee a comprehensive understanding of the dynamic mechanism to generate festival tourist attractions. The meeting notes and interview protocol were established, according to the ethical guidelines of the Helsinki Declaration and were approved by the Human Ethics Committee of Guangdong Mechanical and Electrical Polytechnic (GMEP-2022-01-020). Written informed consent was obtained from individual or guardian participants.

**Table 1:** Details of data collection of Guangfu Temple Fair.

Data collection phase	Data types	Methods	Profile of interviewees
Before the festival	General Scheme, the conference and the interview materials	Grounded theory	7 staff members, including 5 males and 2 females. The average age of respondents was 35 years old.
During the festival	The interview materials	Intertextuality theory	24 tourists, including 13 males and 11 females. The average age of respondents was 32 years old.
After the festival	The interview materials	Grounded theory	21 tourists, including 11 males and 10 females. The average age of respondents was 30 years old.

### 4.4. Data analysis

Constructivist grounded theory suggests that researchers can interpret and define the experience of the research subject as seen in the data based on prior knowledge and experience to understand their own empirical world and the empirical world of the research subject [58]. Using Nvivo12 software, we performed initial coding and focused coding, and completed axial coding by extracting sub-categories through continuous comparative analysis and theoretical sampling. In addition, with the emergence of the core categories, the final story line is formed to ensure the realization of the core grounded theoretical tasks.

Two researchers performed independent initial coding immediately after each interview. During the initial coding process, we named each word, sentence, or data segment recorded from the interviews. To facilitate efficient coding and ensure that the codes fit the data, the initial codes were kept simple and accurate, and we used as many gerunds as possible to preserve the actions, resulting in a total of 189 initial codes. Focused coding is the synthesis and interpretation of large segments of data, which is more directional, selective and conceptual. In this process, we take the most prominent or frequent initial codes or both, and raise them to the level of focusing coding through identification, resulting in 16 focusing codes. In the process of axial coding, initial coding and focused coding were compared and refined, and three sub-categories were developed: 1) Constructing elements of festivalscapes based on festival attraction; 2) Forming live experience at the festivalcapes; 3) Perceiving the festival attractiveness. Based on the information obtained and the resulting concepts, categories and relationships, this study established core categories: "Constructing the festival tourist attraction from the perspective of festival organizers and tourists". Based on Peircean semiotic theory, this study constructs the story line of the semiotic construction of festival tourism attraction. This can be summarized as: In the stage of sign production, festival organizers uphold the concept of attraction and shape the experiential scenario through organizational planning, and tourists interpret the symbols by being in the festivascapes and engaging in cultural, novel, social and emotional interactive experiences with the signs. They believe that festival attractiveness consists of sense of ceremony, distinctive feature, rich activities, and cultural diversity. See Table 2 for details.

**Table 2:** Categorization of nodes.

Category	Sub-category	Focused Code	Initial Code Examples
Constructing the festival tourist attraction from the perspective of festival organizers and tourists	Organizers’ Sign Production: Constructing Elements of Festivalscapes Based on Festival Attraction	Safety Assurance	Strengthening the management of road-traffic order, providing dispersion service; Guiding orderly flow of people
		Cultural Activity	Performing Dragon and Lion Dances; Performing comedy; Planning parade; Displaying of Intangible Cultural Heritage
		Personnel Service	Providing volunteer services; Guidance services; Providing personalized services
		Facility	Inviting Guangfu Internet Celebrities to transmit live shows of the performance tour; Carrying out live performance through new media.
		Creative Interaction	On-line guide and interactions about Guangfu Temple Fair; Planning the content of parent-child activities; Launching intangible cultural heritage creative competition.
		Food	Providing foods, such as snacks, exquisite snacks and souvenir foods
		Trade Show	Example 7: Exhibiting and selling “Dating Happiness” souvenirs during the fair; Display intangible cultural heritage.
		Festival Atmosphere	Displaying the Fliggy artistic device, named “Pearl of the Ancient Path” or “Millennium Pearl,”; Creating multi-sensory atmosphere; Creating lively atmosphere; Designing on-site visual
	Tourists’ Interactive Experiences: Forming Live Experience at The Festivalcapes	Cultural experience	Experiencing the distinctive local culture; Feeling the cultural difference; Enhancing understanding of local culture; Feeling the inheritance of traditional culture.
		Novelty experience	Experiencing creative activities; Discovering some unexpected activities; Feeling the surprise
		Social experience	Increasing interaction with others; Meeting new friends; Celebrating the holidays with others; Improving communication with others
		Emotional experience	Feeling the pleasure; Feeling relaxed; Feeling the beauty of life
	Tourists’ Sign Interpretation: Perceiving the Festival Attractiveness	Sense of ceremony	Experiencing the local festive atmosphere that is different from everyday life; Getting involved with the Wishing Wall; Participating in the parade
		Distinctive feature	Experiencing the local dialect; Learning about local customs; Enjoying a diverse range of souvenirs
		Rich activities	Watching a wonderful show; Tasting rich food; Enjoying new forms of performance
		Cultural diversity	Promoting and passing on traditional culture; Integrating multi-regional cultures; Showcasing local craftsmanship

## 5. Process of semiotic construction of festival attractiveness

### 5.1. Organizers’ sign production

To attract tourists to festivals, organizers often create festivalscapes with strong local cultural features because a proper combination of festivalscape elements enables tourists to immerse themselves in enjoyment [21]. Based on research on the retail service environment, Lee et al. identified seven elements of a festivalscape: convenience, staff, information, program content, facilities, souvenirs, and food [62]. Furthermore, based on prior research, Mason and Paggiaro developed a three-dimensional representation of a festivalscape: pleasure, comfort, and food [21]. A festivalscape reflects the overall environment, which comprises design, aesthetics, lighting, layout, and other elements that promote or stimulate tourists’ cognitive and emotional responses, producing the festival experience [62]. In creating a festivalscape, festival organizers should consider the micro (e.g., the program) and macro (e.g., environmental quality) levels of the festival theme [63]. They should also consider social values and ideals to design flexible and diverse festivalscapes and assign them sign value, which is the charm of a festival as a tourist attraction.

Based on this, this study used as objects of analysis the text, conference and interview materials of the “General Scheme of Guangfu Temple Fair” from previous years. First, we statistically analyzed and compared high-frequency words in the text materials of the General Schemes of 2011, 2015, and 2019, and determined at what point for these years the first commencement, adjustment, and transformation and extensive influence the Guangfu Temple Fair occurred. Second, during the preparatory period, we transformed the General Scheme of 2019 Guangfu Temple Fair and the conference materials into text materials. Subsequently, we employed grounded theory to summarize the elements that constructed the festivalscape, and finally used the interview data from the organizers to test for theoretical saturation. Conclusions were derived on how the festival organizers used the sign production of the festivalscape to construct tourists’ expected experiences.

**Fig 2:**
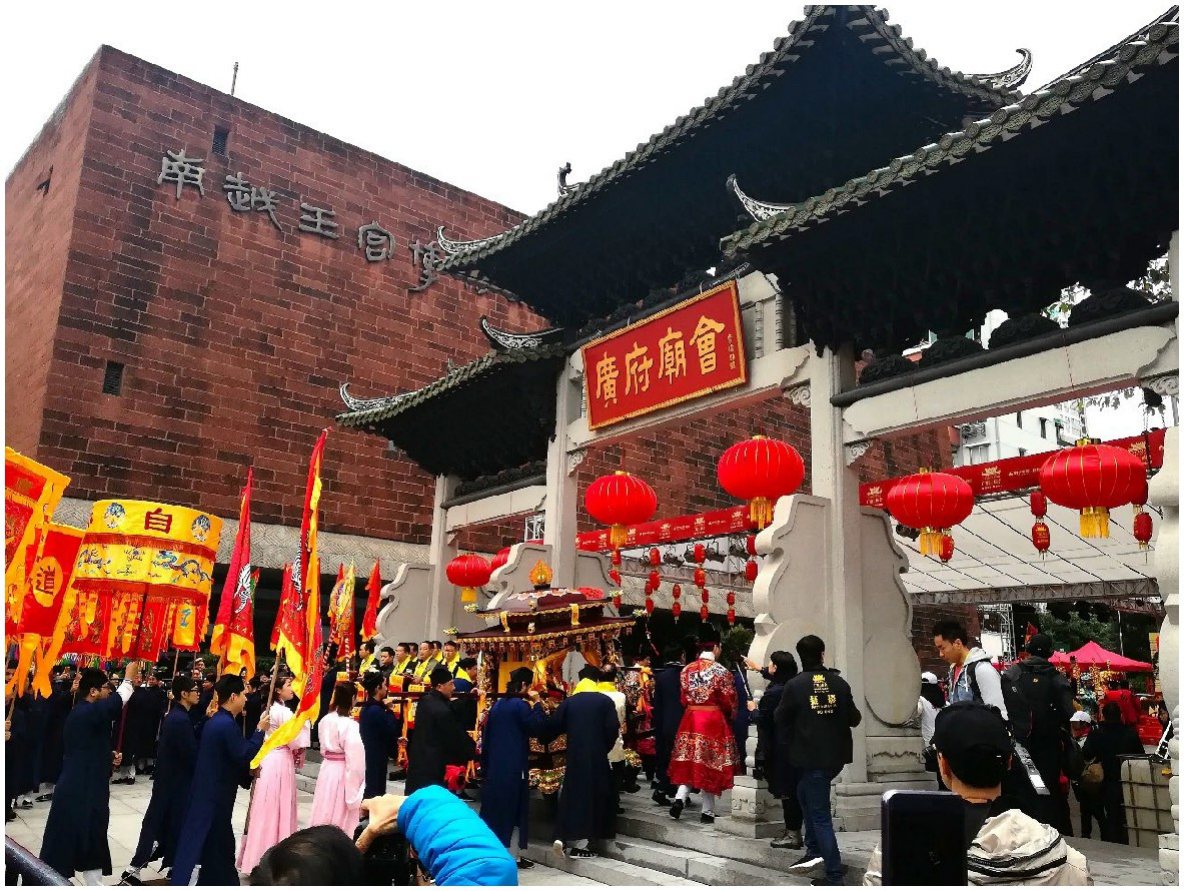
One of the most popular events at the Guangfu Temple Fair: The parade.

**Fig 3:**
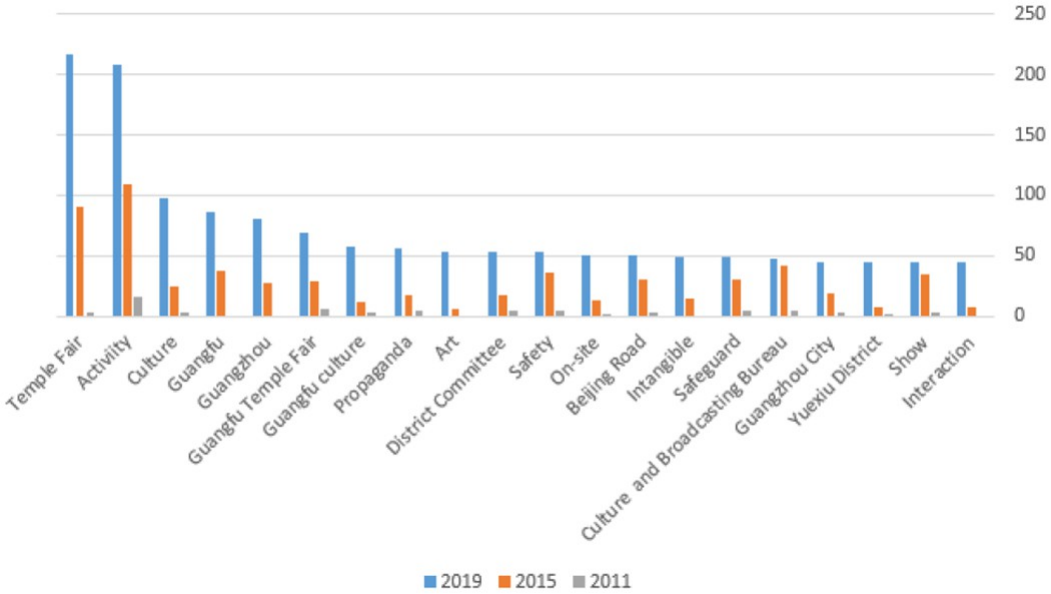
Comparison chart of high frequency words in Guangfu Temple Fair.

Based on the statistics of the high-frequency words in the General Scheme of Guangfu Temple Fair in 2011, 2015, and 2019 (as shown in Fig 2), we excluded high-frequency words irrelevant to this research. Subsequently, we arranged the top 20 high-frequency words according to their frequencies for comparative studies. Fig 3 clarifies that the high-frequency words involve thematic elements that highlight festivalscapes, such as culture, Guangfu, Guangfu Temple Fair, and Guangfu culture. However, they also involve activity content elements that express the festivalscape—temple fair, activity, art, intangible cultural heritage, and exhibition—and security elements that stress the festivalscape—safety, on-site scene, and safeguard. After analyzing the frequency of these words in the overall data and original materials, we found two changes in the three years. First, the frequency of “Guangfu, Guangzhou, art, intangible cultural heritage, and interaction” kept increasing, which indicates that the organizers have gradually realized the importance of Guangfu and intangible cultural heritage and have become more aware of the artistic quality and interactive experience of activities. Second, the frequency of the words temple fair, activity, culture, and others has multiplied, reflecting organizers’ constant innovation concerning activities and exploration of cultural connotations. In general, the organizers of the Guangfu Temple Fair have integrated their understanding of social values and tourists’ expectations into the activities, making the theme clearer and the content closer to tourists’ imagination.

The sign production of festival tourism attraction is actually a process of cultural representation. The practice of representation is the process of concretizing and vectoring concepts and ideas in a sign form that can be communicated and interpreted. The festival tourism attraction itself cannot be the object of direct consumption by tourists, but it needs to be presented to tourists through the carrier of the festivalscape. According to Peircean semiotic theory, the representamen is the carrier of the sign [50]. And whether the festivalscape couples the symbolic meaning represented by the festival tourism attraction and pursued by the tourists depends on whether the festivalscape shaped by the organizer can correctly reflect the characteristics of the festival tourism attraction. Therefore, in the production of festival tourism attraction signs, the organizer fully considers the design of the festivalscape composed of safety and safety assurance, cultural and recreational activity, personnel service, facilities, creative interaction, food, trade show, and festival atmosphere form elements.

Once the festival starts, a large crowd gathers in a short space of time. To avoid conflicts or other disastrous events, safety assurance has become a precondition for a successful festival. Cultural and recreational activities can reflect the uniqueness of the Guangfu Temple Fair and distinguish it from other festivals. Creative interactions strengthen the interactions between tourists and other participants (performers, inheritors of intangible cultural heritage, tourists), enhance tourists’ experience, and deepen the emotional bonds between tourists. Personnel services and facilities including volunteer services, signs, and program activity lists can improve tourists’ satisfaction [64]. Furthermore, as tasting local foods has become a key motivation in the current tourism milieu [65], the organizers strived to provide high-quality foods to highlight the cultural connotation of the “food culture rooted in Guangzhou.” Trade shows combine cultural display and entertainment shopping, strengthening cultural exchanges and promoting economic development through intangible cultural heritage display, Chinese signs, art markets, and so on. Thus, in constructing the festival theme, these tangible factors permeate a strong festival atmosphere. The organizers created a unique space for the festival experience with sound, light, and electricity, and highlighted the festival theme using lanterns, posters, and so on, to create a liminal space that differs completely from daily life. Under the social value of protecting and inheriting the traditional culture and integrating culture and tourism, the organizers created a festivalscape based on their understanding of festival attractiveness and tourists’ expectations, and endeavored to provide tourists with a good festival experience.

### 5.2. Sign interpretation of tourists’ interactive experiences

Through the design and combination of various elements, festival organizers create a festivalscape and then assign meaning to it; thereafter, tourists interpret the festivalscape while experiencing the festival. Tourists’ experience is an interpretation of signs [66]. Guangfu Temple Fair is an experiential activity combining traditional culture with art innovation, which allows tourists to experience the traditional local culture. Here, tourists are attracted by the unique style shaped by physical elements in the festivalscape. Therefore, the festivalscape promotes or stimulates tourists’ cognitive and emotional responses, producing their festival experience [62], which is closely related to the scenes. As embodied entities, tourists interact with the surrounding environment. How their physiological functions interact with the environment determines their tourism experience. Tourists use their senses to interact with elements of the festivalscape, and the interaction produces their physiological experiences that “activate” their psychological feelings [67]. Tourists, festivalscapes, physiological experiences, and psychological feelings constitute an inseparable system, which eventually promotes tourists’ cognition of festival attractiveness.

Under the framework of this festivalscape designed by the organizers, this study identified four festival experience elements through coding and analyzing the transcripts of the on-site interviews with tourists at Guangfu Temple Fair. The four elements are cultural experience, novelty experience, social experience, and emotional experience, which constitute the overall contents of visitors’ on-site experience. First, the organizers’ in-depth exploration of local culture aroused the interest of tourists from different regions. By participating in cultural and recreational activities, trade shows, and food tasting, tourists perceive the festival atmosphere and connect with and understand the local culture, generating cultural experience. Second, through innovation, traditional culture can introduce novelty to tourists, which is both independent and unexpected [68]. As a newly created festival, the 9^th^ Guangfu Temple Fair boldly included innovative elements such as the “AI Robot” and “Online Temple Fair.” These unique elements ensured novel experiences for tourists. In addition, on-site interactive activities can also bring unexpected novel experiences. Third, the festival provided a platform for different groups to interact with each other. Tourists could enhance their bonds with their relatives and friends by participating in various activities and interact with other participants to develop new friendships. Finally, during the festival, tourists immersed themselves in their worlds and gained an emotional experience. Tourists think independently in the liminal space of the festival to gain an emotional experience, which includes aesthetics, relaxation, and being moved. In experiencing the festival, tourists interacted not only with the physical environment but also with the staff, performers, and inheritors of intangible cultural heritage. The cooperation between the senses and body movement promotes proprioception, which helps construct diverse festival experience contents and assign meaning to festival attractiveness following these experiences.

The interview data from tourists collected during and after the festival were analyzed to explore tourists’ (as interpreters) interpretation of festival attractiveness, we found that tourists’ interpretations of festival attractiveness center on four aspects: cultural diversity, distinctive features, rich activities, and a sense of ceremony. Tourists leave their permanent residences to travel to another destination because the destination has attributes that differ from those at their places of residence [69]. These different attributes are reflected in cultural diversity. A festival is a stage where the destination’s culture is shown to the rest of the world, like a miniature version of the local culture. Through collateral observation, tourists can compare differences in cultures and find the uniqueness of a festival.

The customs and culture are remarkably different from where I live. If the city I will go to is different from my hometown, I will think it is unique. But if it wants to attract me, leave a deep impression, and make me like it, it must be better.

—New media man, City God Temple Plaza, Feb. 23, 2019

As the above tourist said, cultural diversity and distinctive features constitute tourists’ initial impression of festival attractiveness and can be considered essential components of attractiveness. However, festivals are temporary cultural activities. More activities are required to attract more tourists during a specific timespan within a particular space. Logically, the richer the activities, the stronger the ability to meet tourists’ needs and the stronger the attractiveness [69]. Further, in a festivalscape that bears local culture, the liminal space of the festival can shape a ceremonial environment. Tourists can share their joy through ceremonial activities such as the opening performance and the Guangfu folk culture performance tour. These elements enhance the sense of participation, enabling tourists to temporarily escape the drill of their daily lives and feel a unique festival ambience.

To sum up, the semiotic construction of festival tourist attractions involves three stages: the representation of festival attraction being produced, tourists interacting with the representation, and tourists assigning meaning to the objects. Specifically, in the sign production stage, based on their understanding of social values and ideals and tourists’ expectations, the organizers created the festivalscape and assigned meanings to it to provide tourists with an attractive festival experience. By coding the general schemes of past years, conference materials during the preparation periods, and interview materials, we found that the organizers had created a festivalscape constituted by the following: security assurance, cultural activity, personnel service, facilities, creative interaction, food, trade show, and a festival atmosphere. This festivalscape condensed the connotation of local culture within its scope and presented it in a way tourists expected. It was flexible, diverse, shapeable, and could be adjusted to conform to tourists’ imagination.

In the interactive experience stage, tourists immersed their senses into the experience of the festivalscape. As a sign medium, the festivalscape connected the organizers and tourists, who used their senses to feel the meaning the organizers had intended to convey. Thus, after analyzing the coding of the in-depth tourist interview texts, we found that tourists’ on-site festival experience could be divided into four types: cultural experience, novelty experience, social experience, and emotional experience. These experiences not only constituted the basis for tourists to “read” the festivalscape but also laid the foundation for tourists to create meaning.

**Fig 4:**
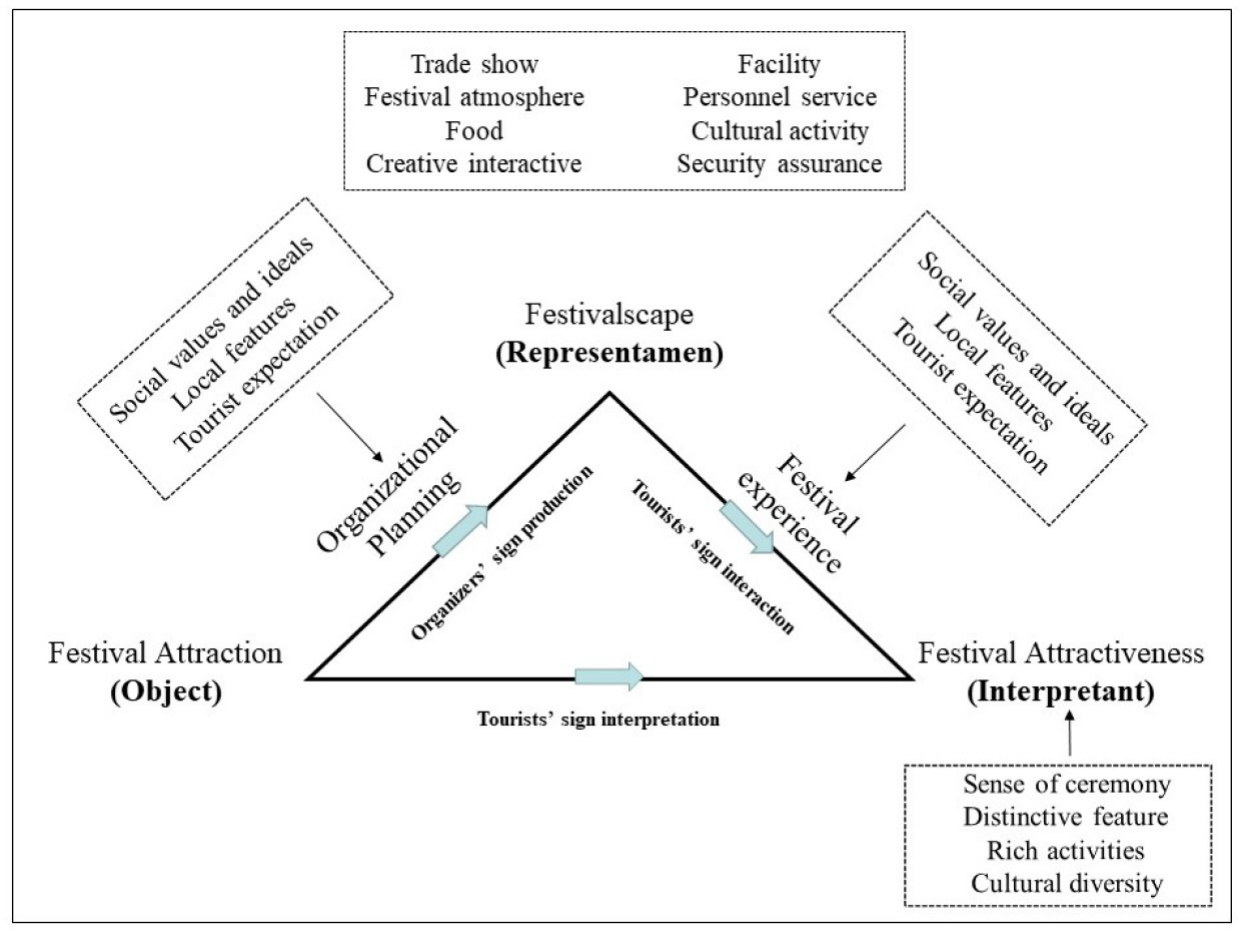
Conceptual model of semiotic construction of festival attraction tourism.

In the sign interpretation stage, tourists tried to understand the meaning organizers had conveyed through active interaction. Simultaneously, the tourists used their collateral observation to assign new meanings to festival attractiveness. They believed that festival attractiveness should be a comprehensive embodiment of cultural diversity, rich activities, distinctive features, and a sense of ceremony. The three stages described above formed a complete semiotic system of the festival tourist attraction, which is open and will continue to develop with the interaction between organizers and tourists. Fig 4 outlines the conceptual model of this three-stage process.

## 6. Conclusions

### 6.1. Research implications

Actual human signs are not reflected in uniformity, but diversity and are not rigid but flexible [70]. The festivalscape represented by a festival as a tourist attraction is just a reflection of such flexibility. As a text, the festivalscape goes through the process in which organizers “write” and tourists “read” it and are involved in tourists’ creation. This study employed the Peircean semiotic theory to elaborate on the semiotic construction process of festivals as tourist attractions. Specific theoretical contributions are as follows.

First, this study enriched the analytical framework of Peircean theory by elaborating the semiotic construction process of festival tourist attractions. Previous research focused on tourist sight attractions [5, 22], neglecting the semiotic construction of festival tourist attractions. Simultaneously, influenced by MacCannell’s ternary relationship of tourist attractions, scholars paid more attention to the signs that provide information about a tourism object [47, 48] but ignored the tourism object itself. Nevertheless, festival tourist attractions differ from landscape tourist attractions in that the festival tourism object has greater instability and malleability. Therefore, in the semiotic construction of festival tourist attractions, the generation of objective and symbolic properties are intertwined. Thus, the organizers shaped both the objective property (the festivalscape) and symbolic property in the same process.

Second, this study adds new insights to the existing literature by exploring the salient dimensions of the festivalscape from the perspective of organizers and visitors. Based on qualitative research, we identified eight dimensions of the festivalscape (trade show, festival atmosphere, food, creative interactive, facilities, personnel service, cultural activity, security assurance). For the organizers, it is important to provide tourists with high-quality content and ensure their personal safety in the festival tourism intensive places, and the safety management of tourists’ health will be very strict; while tourists are more concerned about the variety of experiences brought by the richness of the festival content. Security assurance and festival atmosphere have been neglected in previous studies [21, 62] of festivalscapes. They emphasize the holistic nature of the festivalscapes, reflecting the organizers’ original intention of actively creating a safe and orderly, joyful and peaceful festival atmosphere to meet the spiritual and cultural needs of tourists, as well as the tourists’ expectation to satisfy their emotional needs through the perception of the atmosphere.

Third, the Peircean semiotics theory provided the basis for analyzing the semiotic construction of festivals as tourist attractions. This study used grounded theory to analyze the text materials obtained from case tracking and discussed the empirical results accurately and systematically. Grounded theory plays a crucial role in exploring the dimensions of organizers’ design of festivalscapes and tourists’ perception of festival attractiveness. Moreover, a conceptual model of the semiotic construction of festival tourist attractions was provided, and methodological triangulation was adopted to improve the credibility and validity of the research results [71].

### 6.2. Practical implications

This study not only enriches the theoretical cognition related to semiotic research, but also brings insights to the development and management of festival tourism attraction in terms of application value, with the following aspects.

First, the sign production of festival tourism attractions from the perspective of organizers in this study systematically analyzes the planning and shaping of festivalscapes by organizers, providing a theoretical reference for the development and management of festival tourism attractions in the industry. Organizers can shape attractive festivalscapes, including making every effort to ensure festival safety, arranging various programs and performances, designing creative interactive programs, providing special food and snacks, organizing and holding trade fairs, providing timely staff services, and creating a lively festival atmosphere, to enhance tourists’ participation experience. Specifically, 1) They should continuously improve the diversity and quality of festival activities to immerse visitors in them. 2) They should recognize the importance of food and snacks, and can cooperate with local producers, distributors, processors, hotels and restaurants to provide tourists with delicious and high quality food. If they can’t provide your own food, they can also work with restaurants around the festival to extend the experience. 3) They can use a professional team to design the music, light, color, and smell of the festival site, thus creating a themed festival atmosphere. 4) They can arrange signs and signage highlighting the festival along the route to the festival site, so that visitors can form a good first impression of the festival through careful spatial layout and enter the warm-up for the best experience in advance.

Second, the understanding of tourists’ sign interaction and interpretation process will bring inspiration to the organizer’s organization and management. Organizers should focus on the design of multiple interactive activities, encourage tourists to have beneficial interactions with other participants, and provide tourists with accessible and easy to interact with shared spaces. They can dig deep into local cultural characteristics and traditional ceremony activities, display cultural festival symbols of decoration, ceremony and food system, and organize various festival activities to enhance the participation and interaction of tourists, thus creating a lively and peaceful festival atmosphere. In addition, the organizers should timely understand the quality of tourists’ experience, help tourists to understand the connotation of festival activities through personnel explanation and information release on site, and help tourists to interpret signs more smoothly.

### 6.3. Limitations and further research

This study is a preliminary attempt at applying Peircean semiotics theory in studying Guangfu Temple Fair as a festival tourist attraction. Peircean academic thought is not completely independent, and ideas such as pragmatism philosophy and phenomenology are closely connected with semiotics, however, this study fails to examine them in depth. The subsequent study needs to further optimize and improve the theoretical analytical framework of the semiotic construction of festival tourism attraction formed based on in-depth understanding of Peircean ideological theory. It also combines phenomenology, other scholars’ semiotic ideas (such as Saussure and Barthes) to grasp the symbolic characteristics of festival tourism attraction. Meanwhile, this study mainly takes the Guangfu Temple Festival in Guangzhou, China as a case study, and is committed to revealing the representativeness and typicality of the case, without researching multiple cases to form a comparative case. Festivals cover many types, and future studies can make comparative studies on various types of festival tourism attractions and other types of tourism attractions from the perspective of semiotics.
